# Empowering minorities and everyone in participatory budgeting: an agent-based modelling perspective

**DOI:** 10.1098/rsta.2024.0090

**Published:** 2024-11-13

**Authors:** Dino Carpentras, Regula Hänggli Fricker, Dirk Helbing

**Affiliations:** ^1^ ETH Zurich, Zurich, Switzerland; ^2^ University of Fribourg, Fribourg, Switzerland; ^3^ Complexity Science Hub, Vienna, Austria

**Keywords:** participatory budgeting, collective intelligence, social simulations, agent-based modelling

## Abstract

Currently, there are increasing attempts to better involve citizens in political decision processes. A successful approach in that regard has been participatory budgeting (PB), which allows citizens to propose projects and then decide how to distribute a given budget over them. Meanwhile, the literature on collective intelligence (CI) has also shown the ability of groups to solve complex problems. Thus, by combining CI and PB, it should be possible for citizens to identify problems and create their own solutions. In this article, we study this possibility by using agent-based models. Specifically, we first show that a system combining CI and PB produces solutions that strongly penalize minorities if the solution quality depends on group size. Then, we introduce an approach that can overcome this issue. Indeed, by using a common knowledge base for the storage of partial solutions, the quality of solutions of minorities can benefit from the work of the majority, thereby promoting fairness. Interestingly, this approach also benefits majorities, as the quality of their solutions is further improved by the work of the minorities, thus reaching better solutions for everyone. This stresses the potential and importance of an open innovation approach, which is committed to information sharing.

This article is part of the theme issue ‘Co-creating the future: participatory cities and digital governance’.

## 1. Participatory budgeting and collective intelligence

Participatory budgeting (PB) is an innovation in democratic governance, which offers a possibility for inclusive decision-making processes within communities [1,2]. Originating in Porto Alegre, Brazil, in 1989, PB has since gained traction globally as a means to foster citizen engagement, transparency and accountability in allocating public funds [3].

At its core, PB embodies democratic principles by inviting residents to actively participate in determining how public budgets are allocated. While many different formats exist, most PB initiatives can be divided into two phases. In the first phase, citizens collaborate with local governments to identify community needs as well as possible projects and initiatives. This is usually done through community meetings, workshops and public consultations, where residents can voice their concerns and suggest ideas [4]. Online platforms and surveys are also commonly used to gather input from a broader audience.

In the second phase, the population is called to vote on the proposed projects, some of which are then selected in accordance with the available budget. This is usually done via community-wide voting events, both in-person and/or through online voting systems, ensuring broad and inclusive participation [4].

While PB allows one to achieve legitimate decisions or to strengthen social cohesion [5,6], it is not without challenges and limitations. Indeed, despite its democratic ideals, PB initiatives may still face challenges to be inclusive, particularly with respect to marginalized communities which may lack access to information or face systemic barriers to participation [7].

One of the issues that can affect PB initiatives (as well as many other democratic decision-making processes) is the so-called tyranny of the majority [8,9]. This concept usually refers to the idea that the majority can dominate. Within the context of PB, this refers to the fact that by using common voting systems, the majority could secure a disproportionate part of the budget for their own project. Indeed, the most common voting approach, ‘majority voting’, selects the project(s) that get the largest number of votes until the available budget is used up. In an extreme case, it may happen that one selected project has a cost equal to the entire budget, leaving no budget for any other projects. One may instead decide to select projects not only based on the share number of votes but also by considering the ratio between votes and requested budget, as done in the method of equal shares (MES) [10].

To date, PB has still relied a lot on experts and representatives in many steps of project generation [4], thus limiting how much citizens can affect the final outcome. This seems to be unavoidable, as most projects require some level of expertise. However, citizens can also rely on collective intelligence (CI) to overcome this limitation [11].

Indeed, CI refers to the capacity of groups to make decisions, generate ideas and solve problems more effectively than single individuals [12–14]. CI has garnered increasing attention in recent years, fuelled by empirical studies [11] as well as advances in computational modelling [15–17], social network analysis [14,18] and artificial intelligence (AI) [19–21]. Furthermore, with projects such as Wikipedia and GitHub [22], its practical success has also become evident to the wider public.

Thus, CI could be used to support participatory methods that go beyond voting or beyond consulting citizens for problem identification: citizens can also craft solutions to problems. However, as the literature on CI shows, the quality of results strongly depends on the type of interaction between the people. Indeed, a smartly designed system may be structured such that individual efforts align constructively, producing high-quality results (‘wisdom of the crowds’) [23,24]. In contrast, poorly designed systems may produce chaotic processes and low-quality results (sometimes framed as ‘madness of crowds’) [25,26].

In this article, we explore the possibility of citizens to directly solve their own problems, with a special focus on the implications for minorities. To do this, we rely on abstract models, mainly in the form of agent-based models (sometimes also referred to as agent-based simulations or social simulations).

The remaining parts of this paper are structured as follows: In §2, we discuss how naively merging PB and CI results in inequality, producing results that are much more favourable for large groups. In §3, we introduce a variety of models merging CI and PB, and in §4 we explore them mathematically. Section 5 deepens this exploration using agent-based simulations, and §6 describes some additional variants to verify the robustness of the model. Finally, in §7, we discuss our results and their implications for future studies.

## 2. A new approach to PB

### (a) The problem of group size

Proportional voting methods such as the MES are known to enable a fair sharing of the budget [10]. However, they still do not guarantee fairness in terms of the quality of solutions. To better understand this issue, we can consider the following hypothetical example in which 90% of people like to create a new park and 10% prefer to build a new theatre. Let us call the two groups of people A and B, respectively. With classical voting methods, it is possible that more than 90% of the budget is attributed to the park, leaving little or even no budget for the theatre. A proportional method such as MES aims instead to reserve 90% of the budget for the first project and the remaining 10% for the second one, thus reaching budgetary fairness.

Nevertheless, in a situation where the voting individuals create their own solutions, the number of people involved in the park design is nine times greater than those working on the theatre project. Consequently, group A can develop its project in much more detail. The larger group may even include several experts (e.g. architects, engineers, etc.) to enhance the project’s quality. In contrast, group B has much smaller resources and ‘workforce’, resulting in a theatre design that is probably less detailed and of lower quality.^1^

To understand this problem more clearly, in the following, we analyse it mathematically by exploring how the average utility is affected by the number of people. For this, let us define $u_{g}(X)$ as the average utility that people of group g attribute to solution X . Furthermore, let $C_{X}$ be the cost of project X , and $N_{g}$ be the number of people in group g . Then, we can define the ‘effectiveness’ $E_{X}$ of solution X by the ratio between the total utility and its cost


(2.1) $$\begin{array}{ccc} & E_{X}=\frac{u_{g}(X)N_{g}}{C_{{,}_{X}}}\;. & \end{array}$$

Notice how the effectiveness represents the total utility per unit cost. As we will see, this measure is very important when the budget is proportional to the number of people. Indeed, by using a fair method for budget distribution, the resources available for a project can be assumed to be proportional to the group size. Thus, $C_{x}=\alpha N_{g}$ .^2^ By substituting this into equation (2.1), we obtain


(2.2) $$\begin{array}{ccc} & u_{g}(X)=\alpha E_{{,}_{X}}. & \end{array}$$

In the following, we hypothesize that the more work W is dedicated to improve the solution X , the larger its effectiveness becomes. With ‘work’, we mean the total time spent across the population (i.e. if one person spends 1 h and another one 10 h, the total time is 11 h). Thus, if we suppose that each person works on average β hours, we have approximately $W=\beta N_{g}$ hours of work. This shows how the effectiveness is proportional to the group size, which is important to see that larger groups can accomplish more and, thus, obtain better results.

For the sake of simplicity, we assume here a linear relationships between E , W and $N_{a}$ so that we can write $E=\gamma N_{g}$ . However, as we will show in §6, generalizations or more complex models still produce equivalent results. For example, we will replace the previous proportionality between variables with an exponential relationship. The resulting equation


(2.3) $$\begin{array}{ccc} & u_{g}(X)=\alpha \gamma N_{g} & \end{array}$$

shows that a fair budget (re)distribution is needed, but it is not sufficient to guarantee the fairness of the result. Indeed, we find that the larger the group, the higher the average utility for that group. This can be even better seen by defining an inequality coefficient I . Since, in the following, we will mostly analyse two groups, A representing the majority and B representing the minority, we can define the following inequality measure:


(2.4) $$\begin{array}{ccc} & I=\frac{u_{a}(X_{a})-u_{b}(X_{b})}{u_{a}(X_{a})+u_{b}(X_{b})}. & \end{array}$$

Herein, $X_{a}$ and $X_{b}$ represent the best solutions for each group. When the effectiveness of solutions depends on the group size, we can use equation (2.3), obtaining


(2.5) $$\begin{array}{ccc} & I_{0}=\frac{N_{a}-N_{b}}{N_{a}+N_{b}}. & \end{array}$$

Notice that we use the symbol $I_{0}$ to distinguish it from equation (2.4), which is the general way to calculate inequality, while equation (2.5) represents the case where the utility is proportional to the number of people in a group. If the size of both groups is equal, we have $I_{0}=0$ , while the value of $I_{0}$ is approximately 1 if $N_{a}\gg N_{b}$ . In the following, we study different models in situations where $N_{a}\gg N_{b}$ .

### (b) Toward making solutions fair

Many possibilities exist for restoring the fairness of solutions and thus bringing I close to zero. However, most of them end up introducing new imbalances to the process. For instance, one may limit the budget of larger groups, but this would be restrictive and potentially perceived unfair in terms of budget allocation. Furthermore, this approach may eventually result in strategies where large groups divide themselves up into smaller groups to obtain more budget. Others may think of forcing large groups to spend some of their time contributing to the projects of smaller groups. However, this approach would again substantially interfere with large groups as it would take away some of their time. It would also probably undermine participation altogether, as activities such as PB are heavily based on voluntary work. Forcing people to perform tasks they are not interested in would probably be counterproductive.

In this article, we aim to develop a win–win strategy, thus, a system that can help minorities but not at the cost of larger groups. Indeed, as we will observe in later sections of this manuscript, even majority groups can benefit from the presence of minorities. To achieve this, we make use of the fact that many solutions share common elements. For instance, the design of a pedestrian pathway may be common to many different city-level projects. Similarly, many buildings share some common features such as doors and staircases, and many projects have to satisfy the same legal requirements. This means that parts of a project could be re-used in other projects if an open innovation approach is pursued.

Notice, however, that some parts can be used in the exact same way (e.g. door design could be used multiple times), while other parts may require some level of fine-tuning. For simplicity, until §5, we will not analyse this distinction but focus instead on the overall benefits that re-use of solution elements can have.

Thus, in the following, we explore the introduction of a shared knowledge base, in which solutions are stored for re-use in other projects. In later sections, we explore different versions of such a shared knowledge base and how it can be designed to simultaneously promote high-quality solutions and fairness.

## 3. Model

In this section, we propose an agent-based model of how CI can be integrated into PB. This allows us to produce a stylized version of how people may interact in such a system. Indeed, agent-based models make it possible to explore different types of interactions, both mathematically and by means of simulations. Notice, however, that owing to the complexity of the subject, the model may appear stylized, as it simplifies many aspects of human behaviour and idea creation. Such a stylized approach is usually taken in these kinds of models to keep them tractable [31]. Indeed, when trying to make a model more realistic, one often needs to sacrifice explanatory power (interpretability) owing to the many variables and parameters involved. This often results in well-known problems, such as high-parameter sensitivity and the black-box problem typical of many AI models.

The model we study represents the behaviour of two groups, A and B, across multiple PB initiatives in a simple way. Every solution initiative is uniquely identified with the time variable t so that the first initiative will take place at t=1 , the second at t=2 , etc. Furthermore, every initiative consists of an idea creation phase and a voting phase. In the idea creation phase, each group g tries to develop a project $p_{g,t}$ with a solution $X_{g,t}$ that maximizes the group’s average utility $u_{g,t}(X_{g,t})$ . Practically, this means that the project each group is working on changes in each time step (i.e. with each initiative) and, thus, also the utility function and best possible solution.

For now, to keep the model simple and the results easy to interpret, we assume that every person within the same group shares the same individual utility function. Thus, the individual utility function coincides with the average utility function of the entire group. This also means that in the voting phase, all agents belonging to the same group g vote for the same best solution $X_{g,t}$ . Let us further assume that the budget available to each project is proportional to the number of people supporting it.^3^ These assumptions allow us to focus on the dynamics of the idea generation phase, while in §6, we will generalize the model by testing more complex alternatives. This will make it possible to confirm that the simplifications we have made here do not affect the overall conclusions from the simplified model.

We define every solution $X_{g}$ to be a point in a D -dimensional space so that $X_{g}=(x_{1},x_{2},\ldots ,x_{D})\in {\mathbb{N}}^{D}$ . Every dimension here represents the domain of a subsolution. For instance, if the problem concerns creating a park, one of the dimensions may represent different pathway designs, another dimension is the design of an artificial lake, etc.

The value of D represents the set of all dimensions for all possible projects, while not all projects may depend on all dimensions. For example, usually, a theatre will not need an artificial lake. For simplicity, we further assume that every project depends on δ<D dimensions and write the overall utility as


(3.1) $$u_{g,t}(x_{1},x_{2},\ldots ,x_{D})=\sum_{d=1}^{D}w_{g,t,d}\cdot f_{g,t,d}(x_{d}).$$

Herein, $w_{g,t,d}$ is zero if the project does not depend on dimension d and 1/δ otherwise. Instead, $f_{g,t,d}$ is a function linking the utility to the subsolution $x_{d}$ . This class of functions should be such that each function is independent of each other. Even if each x is modelled as a natural number, this value only represents a unique identifier, representing the solution that was produced first. Furthermore, since the value of x does not represent similarity, two solutions $x_{1}$ and $x_{2}$ may be very close numerically, while still producing quite different values of $f_{g,t,d}(x)$ . The final assumption regarding the f functions is that the average utility of the best solutions linearly increases with the size of the explored space (in §6, we test some alternatives to this relationship). Accordingly, here we have the equation


(3.2) $$\begin{array}{ccc} & E[\max\;f_{g,t,d}(x)]=\lambda x_{{,}_{M}}, & \end{array}$$

where $x_{M}$ is the maximum value of x (assuming that the exploration started at x=1 ). To respect all these conditions, we specify each $f_{g,t,d}(x)$ according to


(3.3) $$f_{g,t,d}(x)=\min\left(\frac{1}{v},x_{\;M}\right).$$

**Figure 1.**
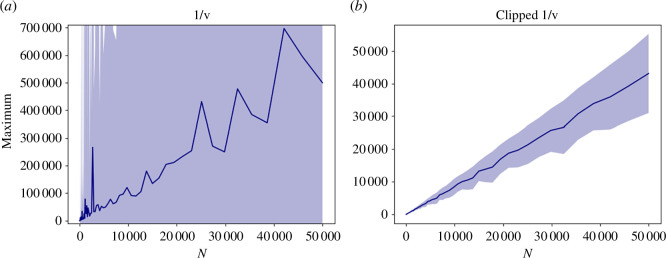
Expected maximum over N trials for (*a*) 1/ v and (*b*) 1/ v clipped, as shown in equation (3.3).

Here, v is a uniformly distributed random variable in the interval (0,1]. In figure 1, we show why we included the minimum in equation (3.3) instead of simply modelling f as 1/v . Indeed, as shown in figure 1*a*, already 1/v satisfies equation (3.2). However, such a distribution is characterized by an extremely large variance, where the standard deviation is more than 25 times as large as the mean value. Computationally, this is a problem as it introduces very large variability in the results. Moreover, this is also highly unrealistic, as it implies the possibility of finding outstanding solutions in just a few steps. To overcome such problems, we introduced a minimum function in equation (3.3). As one can observe in figure 1*b*, this still implies a linear increase (thus satisfying equation (3.2)), while keeping a reasonable level of variability. In this case, however, the standard deviation is only 0.27 times the mean value.

### (a) Structure of the model

To better understand the properties of the co-creation system we are interested in, we will study and simulate different agent-based models. Despite the differences between models, however, they still have similar structures such that we can define a base model $M_{0}$ and discuss the other models as its variants.

In model $M_{0}$ each group has a short-term knowledge base $k_{g}$ . This is a repository where the subsolutions $x_{d}$ are stored, and it will be the only repository used by agents during the idea creation phase. This short-term knowledge base is complemented by a long-term one $K_{g}$ . The main difference between the two is that $k_{g}$ starts empty at the beginning of each initiative (thus erasing previous information), while $K_{g}$ will indefinitely preserve everything that is stored in it.

Our model simulates a total of T initiatives. For each initiative, the following steps are followed:

- *Project identification*. For each group, δ domains are selected out of the D possibilities. These will remain the same until the initiative is completed. These are selected independently from previous initiatives and from other groups.
- *Knowledge base exploration*. Each short-term knowledge base $k_{g}$ starts empty. In contrast, the long-term knowledge base $K_{g}$ may contain previous solutions from previous rounds. If this is the case, all solutions relevant to the current dimensions are copied into the project’s knowledge base $k_{g}$ , and the respective values of $f_{g,t,d}(x_{d,i})$ are calculated for all the solutions $x_{d,i}$ .
- *Idea generation*. Every agent generates n new solutions on average. This is done by repeating the following loops nN times (where $N=N_{a}+N_{b}$ is the total number of agents):
  - Select an agent with a probability proportional to their group’s size $N_{g}$ (accordingly, larger groups will be selected more often).
  - Let the agent choose a domain from the ones relevant to the project of their group.
  - Let the agent produce a solution $x_{d,i}$ for domain d .
  - Calculate the value of $f_{g,t,d}(x_{d})$ .
  - Add the solution $x_{d,i}$ to the short-term knowledge base of the group. Note that new solutions may also be interpreted as effort to fine-tune previous solutions.
- *Project selection*. For each group, select the best solution $X_{g,t}$ from their own short-term knowledge base $k_{g}$ .
- *Knowledge base update*. Copy the δ subsolutions $x_{d,i}$ , which compose the final solution $X_{g,t}$ , from the project’s short-term knowledge base $k_{g}$ to the long-term knowledge base $K_{g}$ .

Note that this formulation does not explicitly mention costs, as we assume that the budget available to a project is proportional to the number of people voting for it (as discussed in §2*a*, $C_{x}=\alpha N_{g}$ ). Furthermore, since for simplicity we assume that all the $N_{g}$ people vote for that project, we have a proportionality between the available budget and the number of people voting for a project. Therefore, explicitly adding cost and a voting phase would only slow down the simulation without adding any interesting dynamic behaviour. Indeed, for every initiative, the cost of project $X_{g}$ would be exactly $\alpha N_{g}$ , and this project would be voted by the $N_{g}$ people of group g , and thus always being selected.

In the next sections, we analyse four variants of this model:

— *M1: Separate knowledge bases and no long-term knowledge base*. This model represents the case in which groups work independently and store no information for later rounds. So the only difference to $M_{0}$ is that step (v) is removed such that the long-term knowledge base is always empty.
— *M2: Separate knowledge bases plus long-term knowledge base*. In this case, every group works with different knowledge bases, but they store all partial designs for future rounds. Thus, in this model, instead of copying only the final, best design, in step (v) they copy everything from the short-term knowledge base $k_{g}$ into the long-term knowledge base $K_{g}$ .
— *M3: Common knowledge base containing final designs*. This model differs from $M_{0}$ only in the aspect that groups do not maintain separate knowledge bases but instead a shared one.
— *M4: Common, long-term knowledge base with all designs*. In this case, both groups share their long-term knowledge base and also store all partial designs.

While the point of this study is not to produce a fully realistic model, these simplified models still allow us to explore how different knowledge bases and kinds of interactions affect the final outcome. We would further like to highlight that models M1 and M3 are ‘optimality-oriented’ in the sense that they are mainly focused on the final, optimal solution for the current initiative. In contrast, M2 and M4 have a more ‘evolution-oriented’ approach, where many alternatives are kept. Indeed, this resembles ecosystems, which are not dominated by a single species, but based on the presence on multiple different species. In a similar way, we note that M1 and M2 are based more on a competitive approach, while M3 and M4 are based on sharing knowledge. In the next sections, we study these models using both, mathematical analysis and computer simulations.

## 4. Mathematical exploration of the model variants

Combining equations (3.2) and (3.3), the expected value of the best solution (represented by ${\hat{u}}_{g,t}$ ) is proportional to the size of the explored solution space, hereafter represented by $L[k_{g}]$ . This term can be further broken down into the designs that were imported from the long-term knowledge base $K_{g}$ and the ones which, instead, are produced during the current initiative. Let us call them $L_{l,g}$ and $L_{s,g}$ , respectively. Then, we have


(4.1) $$\begin{array}{ccc} & {\hat{u}}_{g,t}=E[\max[u_{g,t}(X_{g})]]=\frac{\lambda }{\delta }L[k_{g}]=\frac{\lambda }{\delta }(L_{l,g}+L_{s,g}). & \end{array}$$

While a detailed discussion of the model results, based on agent-based simulations, is presented in the next sections, here we explore how the different models are expected to behave for large values of t (i.e. in the limit of many initiatives).

Notice that model M1 does not require any approximation. Indeed, we have $L_{l,g}=0$ , and $L_{s,g}$ is always equal to $nN_{g}$ . Thus, in model M1, the utility is proportional to the number of people in a group: ${\hat{u}}_{g,t}=\lambda /\delta nN_{g}$ . Consequently, the related inequality coefficient coincides with the benchmark value $I_{0}$ .

In model M2, every initiative produces $nN_{g}$ solutions over δ dimensions. The main complexity is introduced by the fact that some dimensions may be selected more often than others. However, in the approximation of large values of t , for every dimension, we can assume that approximately $nN_{g}t/D$ solutions have been produced over the past t initiatives. To obtain this number, we consider that $nN_{g}t$ solutions x are produced over t initiatives and divide this value by the total number of dimensions D . As the long-term knowledge base contains δ dimensions that are relevant to the current project and they have been produced until t−1 , we can write $L_{l,g}=\delta \;n/D\;N_{g}(t-1)$ . To this, one should add the $nN_{g}$ new designs in initiative t , thereby obtaining


(4.2) $$\begin{array}{ccc} & L[k_{g,t}]=\frac{\delta }{D}nN_{g}(t-1+\frac{D}{\delta })\approx \frac{\delta nN_{g}t}{D}\;. & \end{array}$$

Note that in the last term, we have simply applied t≫−1+D/δ .

One can see that the utility of each group increases from $nN_{g}$ in model M1 to $\delta nN_{g}t/D$ in model M2. Furthermore, in M2 both groups a and b produce solutions which progressively become better and better as t increases. However, in both models, the utility is proportional to the group size $N_{g}$ , thus still resulting in an inequality level of size $I_{0}$ .

In contrast to models M1 and M2, models M3 and M4 are based on the idea of a shared knowledge base. For model M3, the two groups add only their final solution to the knowledge base so that $L_{l,g}=2\;\delta t/D$ (as only δ solutions are added each time by each of the two groups). By applying the approximation of large values of t again, we find that the number of solutions in the long-term knowledge base is much larger than the number of solutions produced in the last initiative (i.e. $L_{l_{g}}\gg L_{s_{g}}$ ). With this approximation, we find that the utility of the two groups is identical, bringing the inequality down to zero. While this is a great advancement in terms of fairness, it also represents a step backward as compared to M2, since the utility values are now much smaller, that is, by a factor $nN_{g}$ . Thus, the approach of model M3 achieves fairness at the cost of slowing down the entire problem-solving process.

Finally, let us consider model M4. In this case, for large t , the utility of the best design ${\hat{u}}_{g,t}$ depends uniquely on $L_{l,g}$ . However, $L_{l,g}$ does not depend on the number of people in the group $N_{g}$ but on the total number N of people in the entire population. Indeed, $L_{l,g}=\delta nNt/D$ . This result combines the favourable features of models M2 and M3, while outperforming each of them. Similar to M3, the inequality value is zero, and similar to M2, the utility is proportional to the number of people. Since we designed the entire system to decrease inequality, it is expected that the utility of the minority group is much larger than in the other models. Indeed, the minority benefits a lot from the work of the majority. Interestingly, however, the situation is also improved for the majority group, which benefits from the work conducted by all minorities, as the utility of each group is proportional to the size of the entire population. We list all these results in table 1.

Note that even in case where the majority represents as much as 90% of the population, moving from M2 to M4 produces a significant performance increase of 10% for the majority. In the more common case, where the largest group represents less than 50% of the population while the remaining people are distributed over many minority groups, moving from M2 to M4 allows the largest group to achieve more than twice the utility it can produce by itself.

## 5. Model exploration with agent-based simulations

The use of agent-based (computer) simulations allows us to study the four different models in more detail without imposing approximations or constraints. However, to run the simulations, we have to choose certain parameter values, by which we lose some of the generality that an analytical mathematical analysis offers. We start by simulating 1000 agents, where 990 of them belong to group A, while the remaining 10 people constitute group B. This ratio has been chosen such that the benchmark inequality value $I_{0}$ is close to 1 (precisely $I_{0}$ = 0.98).

**Figure 2.**
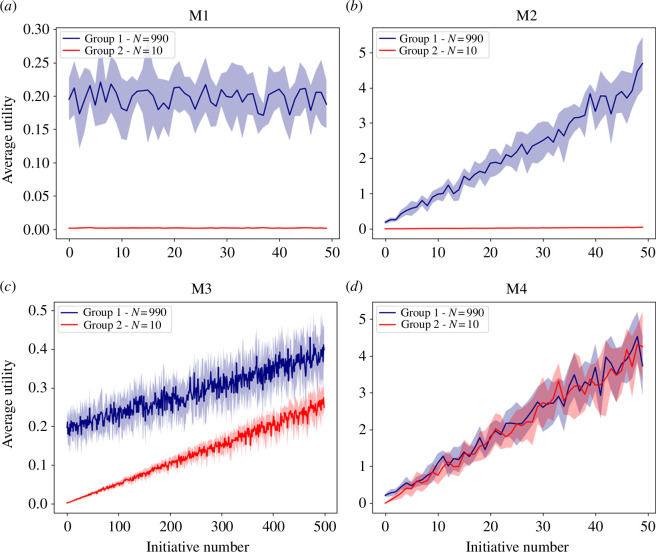
Simulated utility for the majority (blue) and the minority (red) in all four models, shown over time. Solid lines represent the mean, while the shaded area represents the standard deviation. The respective simulated models are (*a*) M1, (*b*) M2, (*c*) M3 and (*d*) M4.

**Figure 3.**
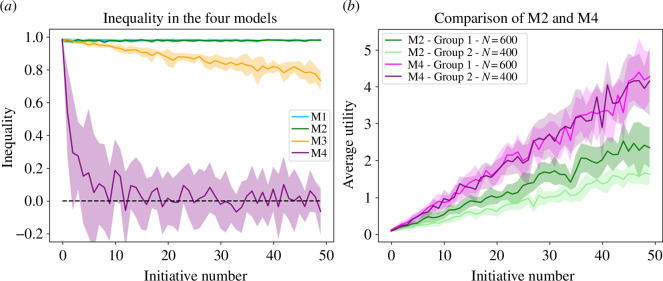
(*a*) Inequality in the four models. (*b*) Comparison of model M2 (represented in green) and model M4 (represented in purple) when the two groups represent 60% and 40% of the population. Solid lines show the mean value and the shaded area the standard deviation.

In the following, we assume D=10 possible dimensions, while δ=3 of these dimensions matter for each solution. We set n (the average number of solutions per person) equal to 10. Since our simulations include random elements, we simulate every configuration 100 times and display the results for the utilities in figure 2 and the inequality values in figure 3*a*. As expected, M1 represents a case in which there is no growth (i.e. no learning) from one round to another, while the inequality value is very close to 1. In model M2, each group learns from their own previous successes, allowing the majority to make quick progress, which moves the utility from values close to 0.2 (as produced by M1) to more than 4 in 50 rounds. However, the minority group progresses much slower, which keeps the inequality value close to 1.

**Table 1.** Comparison of the different models and their utility changes over time (for the approximation of large t ). Notice that model M4 is able to outperform all models in terms of the utility ${\hat{u}}_{g,t}$ , while getting the inequality level down to zero, thereby maximizing fairness.

model	expected utility of best solution ${\hat{u}}_{g,t}$	inequality I
M1	$\left(\gamma /\delta \right)\,n\,N_{g}$	$I_{0}$
M2	$\left(\gamma /D\right)\,n\,N_{g}\,t$	$I_{0}$
M3	(δ/D)⁢t	0
M4	(γ/δ)⁢n⁢N⁢t	0

Since model M3 progresses very slowly compared to the other models, we display our results up to T=500 , while all other models are shown up to T=50 . Indeed, even after 500 rounds, the maximum utility is still 10 times smaller than for model M2 at T=50 . As observed before, model M3 can reduce the inequality on the long run, but it progresses slowly and penalizes the majority compared to model M2.

**Figure 4.**
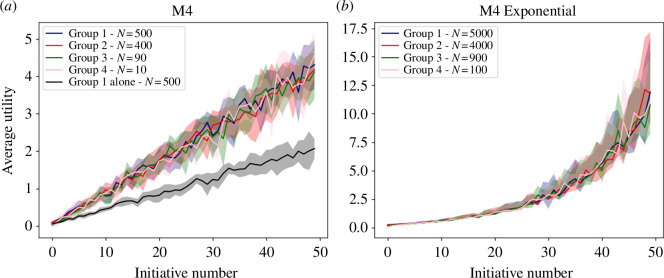
Simulations of model M4, adding personal variations of utility, for (*a*) four groups of different size (colours) in comparison with the largest group working alone (black) and (*b*) four groups in the case of exponential increase.

Finally, model M4 shows results for the utility that compare well with model M2, while also reducing the inequality quickly. Indeed, the inequality values are close to zero from approximately the 10th initiative onward. In figure 4*b*, we also show how models M2 and M4 perform in a case where the majority represents 60% and the minority 40% of the population. Since the two group sizes are quite similar, then, in model M2, we do not observe large differences between both groups’ utilities anymore. However, M4 allows the two groups to benefit from each other, thereby largely outperforming the utility values achieved by model M2.

## 6. Exploration of model alternatives and robustness

In the previous sections, we have focused on studying two homogeneous groups, in which each agent had exactly the same value of utility. In this section, we repeat the previous analysis of model M4 but instead with four groups of sizes 500, 400, 90 and 10. Furthermore, we now assume that the values of the utility functions are different for each agent. Specifically, we introduce a function f that also depends on the agent i , thus being written as $f_{g,t,d,i}$ . Since $u_{g}$ is the average utility function, considering equation (3.1) we assume $f_{g,t,d}$ to be the average of $f_{g,t,d,i}$


(6.1) $$\begin{array}{ccc} & f_{g,t,d}(x_{d})=\frac{1}{N_{g}}\sum_{i}f_{g,t,d,i}(x_{d}). & \end{array}$$

Concretely, we model each $f_{g,t,d,i}(x_{d})$ as a normally distributed function with a mean value of $f_{g,t,d}(x_{d})$ and a standard deviation of 1.

Results are presented in figure 4, where we show the utility of the four different groups (in colour) compared to the performance when the majority works entirely for itself (in black). Our results show that when all groups share their results, they are able to achieve similar levels of utility, thus bringing the inequality down to zero. In addition, every group benefits from the results produced by each other group, as we can see from the fact that they all exceed the performance of the largest group when it works in isolation (black line).

**Figure 5.**
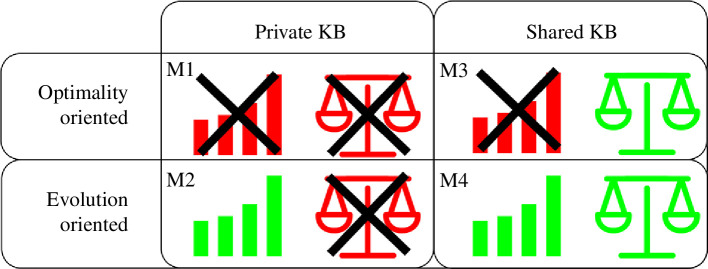
Schematic summary of the results from the four models. Depending on the choices regarding the knowledge base (KB) and whether results are shared between groups or not, we find four different outcomes: (M1) low-quality results and inequality, (M2) high-quality results and inequality, (M3) low-quality results with fairness and (M4) high-quality results and fairness.

To test even more the generalizability of results, we can notice that the simulations presented above made the following assumptions:

— the number of agents was fixed to 1000,
— the number of relevant dimensions was fixed to δ ,
— old designs could be re-used without any penalty or costs, and
— the number of solutions in the knowledge base and the quality of the best solution were assumed to be proportional to each other.

To show that our results are not limited by these assumptions, we changed them by assuming:

— 10 000 agents,
— a random δ ranging from 2 to 5,
— a penalty of 10% on the utility of previous designs (while designs produced during the current initiative will have no penalty), and
— an exponential relationship between the number of solutions in the knowledge base and the quality of the best solution.

The results in figure 4*b* still show that the utility of all groups keep growing in the same way. The main difference is that the shape of the curve is now exponential rather than linear, but M4 is still able to produce fairness in terms of utility. Indeed, the mean value of the inequality coefficient (calculated along the different time points) is 0.06 ± 0.24. Additionally, we repeated the simulation using instead a logarithmic function, which produced an average inequality of 0.06 ± 0.23, thus confirming that the ability of model M4 to guarantee fairness is not due to a specific choice we have made but to the properties of the model itself.

## 7. Discussion

In this article, we have discussed the possibility of using CI in PB. In particular, we have studied four different co-creation scenarios, for which we found (see figure 5):

— *Low efficiency and inequality (in model M1)*. This happens when groups do not share their solutions and do not save them for later use.
— *High efficiency and inequality (in model M2)*. This happens when groups save their solutions for later (re-)use but do not share them with other groups.
— *Low efficiency and equality (in model M3)*. This results when groups share information among each other, while saving only their final results.
— *High efficiency and equality (in model M4)*. This results in preserving and sharing all partial solutions (design results).

A particularly favourable result of model M4 is that it does not only perform highly, while allowing to quickly reduce inequality. It also produces a win–win situation, where every group, independent of its size, benefits from the results obtained by every other group. This is in stark contrast with scenarios where large groups are burdened by having to help minority groups. We would further like to point out that such a win–win set-up could promote social cohesion and, thereby, help overcome polarization [32]. Furthermore, the so-called IKEA effect [33], according to which individuals value things they helped build or create more highly than other products, could significantly promote the adoption and appreciation of solutions derived from the participatory process underlying model M4. The expected reduction of inequality is also well aligned with the United Nations resolution on trustworthy AI, which aims at closing the digital divide [34].

Of course, we are aware that there are many additional research directions that future studies may explore. For instance, one might investigate the role of expertise. It may be important to distinguish between a design developed by an expert and one developed by a person with basic knowledge on a topic, but the availability of generative AI may increasingly help close this knowledge gap. Similarly, the level of detail of a solution could be considered.

Another potential limitation is that this study focuses on stylized analytical and agent-based models. Future studies may explore more realistic models (e.g. following the KIDS principle [31]) or even perform lab or real-world experiments. It would be particularly interesting to develop models that can directly be used with empirical data [35], which would require developing a model and a series of experimental measurements that are compatible with each other.

Overall, however, this study has shown good reasons to believe that PB can strongly benefit from CI. Non-hierarchical approaches, such as those we have studied here, allow people to be involved in the decision-making processes while contributing their own solutions. Furthermore, the possibility of win–win solutions offers the possibility of not only empowering minorities but also even benefiting majority groups. As we continue to refine and test these models, both in theory and practice, we are optimistic that such approaches will help pave the way for more innovative governance structures, fostering greater social cohesion and trust in the process.

## Data Availability

All the code can be found in the following repository: [36].

## Footnotes

^1^
Note that the architectural examples are mainly for illustration of the remixing concept—we realize the simplifications, limitations and caveats of them. Still, historical styles and the history of architecture in general may be considered to be a result of copying, reinterpreting and being influenced by design ideas, motifs and traditions, even though this is not always acknowledged [27,28]. Moreover, some co-created architectures actually do already exist [29]. Also, note that a co-creation approach is strongly advocated for by the ‘open-source urbanism’ movement Bradley. Furthermore, ‘Kreyon City’ was an experiment aiming to demonstrate the potential of co-creation for city making [30].

^2^
The reader may also notice that, here, for simplicity, we are assuming the cost to be *exactly* proportional to the number of people. Instead, in real systems such as in MES, there may be some minor deviations from exact proportionality, even if on average proportionality still holds.

^3^
Note that our model, following the KISS principle, simplifies the complexity of the real process. In accordance with this, exact proportionality seems to be the simplest plausible assumption. Here, this does not assume the application of the MES or any other specific method of (re)distributing resources, here.
